# The clinical and phenotypic spectrum of PTEN hamartoma tumor syndrome: a retrospective cohort study

**DOI:** 10.1007/s10689-026-00604-w

**Published:** 2026-09-17

**Authors:** Einat Ritter, Aasem Abu Shtaya, Revital Kariv, Sari Lieberman, Elizabeth Half, Maria Postnikov, Guy Rosner, Hana Strul, Nathan Gluck, Lior Katz, Ephrat Levy-Lahad, Dalit May, Ido Laish, Yael Goldberg, Elez Vainer, Zohar Levi, Maya Aharoni Golan

**Affiliations:** Department of Gastroenterology and Liver Diseases, Tel Aviv Sourasky Medical Center, Tel Aviv, Israel

**Keywords:** PTEN hamartoma tumor syndrome (PHTS), Cowden -syndrome, soft tissue malignancy, Mosaicism

## Abstract

PTEN Hamartoma Tumor Syndrome (PHTS) is a rare condition characterized by a complex phenotype including gastrointestinal hamartomas and increased lifetime malignancy risk. To characterize the clinical phenotype, genetic landscape, and spectrum of malignancies within a large cohort of adult PHTS carriers. A retrospective cohort study of adult patients (≥ 18 years) followed at specialized high-risk clinics who met PHTS diagnostic criteria based on either: (1) identification of a germline PTEN pathogenic/likely pathogenic variant, or (2) fulfillment of clinical criteria according to National Comprehensive Cancer Network (NCCN) guidelines. A total of 62 individuals were included (62.9% male; median age at inclusion 39.5, IQR, 28–52). All fulfilled diagnostic criteria for PHTS. *PTEN* pathogenic or likely pathogenic variant were identified in 50 individuals (80.6%). Colonic polyps were present in 40 patients. Malignancies were reported in 61% (n = 38), most frequently breast (n=13) and thyroid (n=7). Three individuals (4.8%) had rare soft tissue tumors: one with a desmoid tumor at age 18, one with concurrent axillary osteosarcoma and bilateral chest liposarcoma, and one with a skull-base sarcoma. Macrocephaly was observed in 33.8% (n = 21), and developmental delay, including autism spectrum disorder, in 8 patients (12.9%). One case of *PTEN* mosaicism was identified through tumor sequencing after negative blood genetic testing. Five individuals (8%) were treated with Sirolimus. This large cohort highlights the broad clinical spectrum and substantial cancer burden in PHTS, including rare soft tissue tumors. Constitutional mosaicism should be considered when clinical suspicion persists despite negative germline testing.

**Supplementary Information:**

The online version contains supplementary material available at https://doi.org/10.1007/s10689-026-00604-w.

## Introduction

The PTEN hamartoma tumor syndrome (PHTS) encompasses a spectrum of rare disorders caused by a germline pathogenic/likely pathogenic variant (P/LP variant) within the phosphate and tensin homolog (*PTEN*) gene (MIM 601728). *PTEN*, located on chromosome 10, functions as a tumor suppressor gene regulating cellular growth. Beyond its role in suppressing tumorigenesis, *PTEN* is crucial for normal embryonic development, particularly within the central nervous system where it orchestrates neural stem cell proliferation, cell migration, and synaptic plasticity. A pathogenic or likely pathogenic germline variant leads to dysregulation of the mTOR pathway, which normally promotes cell growth, survival, and proliferation, thereby enhancing cell proliferation and inhibiting apoptosis [1]. In addition to its role in tumorigenesis, *PTEN* loss has been increasingly linked to immune dysregulation and chronic inflammatory processes. This is mediated through aberrant activation of the PI3K–AKT–mTOR signaling pathway, which influences cytokine production, immune cell differentiation and the tumor microenvironment [2].

PHTS encompasses several historical clinical subtypes, such as Cowden syndrome and Bannayan-Riley-Ruvalcaba syndrome, which are now recognized not as distinct syndromes, but as a single disease entity with age-dependent phenotypic presentations that can occur within the same family [3, 4]. The prevalence of PHTS is estimated at 1 in 100,000 to 200,000 individuals. While childhood presentations are typically dominated by macrocephaly, autism, developmental delay, vascular malformations, or benign intestinal polyposis, cancer-related features become prominent in adulthood. Adults with PHTS are highly predisposed to both benign and malignant tumors, most notably breast, thyroid, and endometrial cancers, as well as renal cell carcinoma,colorectal cancer and melanoma. A pathogenic or likely pathogenic germline variant in *PTEN* drives these diverse manifestations across the lifespan [5–7].

PHTS diagnosis is based upon diagnostic criteria, including the most recent National Comprehensive Cancer Network (NCCN) guidelines that recommend relying on a combination of major and minor clinical criteria or the detection of a *PTEN* pathogenic or likely pathogenic variant for definitive diagnosis [8]. Despite established diagnostic criteria, the wide phenotypic variability of PHTS poses significant challenges in identifying affected individuals. While some patients present with only mild or benign manifestations, others experience developmental delays, severe clinical symptoms, or life-threatening malignancies. Furthermore, uncommon or atypical features remain poorly characterized, complicating both diagnosis and counseling [9].

The aim of this study is to describe the spectrum of the clinical, endoscopic, and genetic characteristics of patients with PHTS with an emphasis on rare clinical findings and diagnostic approach.

## Methods

This retrospective cohort study included adult patients (age ≥18 years) who were diagnosed with PHTSbased on either: [1] identification of a germline PTEN pathogenic/likely pathogenic variant, or [2] fulfillment of clinical criteria according to National Comprehensive Cancer Network (NCCN) guidelines.

A clinical diagnosis was established based on the presence of a combinations of major and minor criteria.[10] Patients were followed in six tertiary academic centers. Age at diagnosis was defined as the chronological age at which the patient was first clinically or genetically diagnosed with PHTS. Age at inclusion was defined as the age of the patient at the time of study initiation and cohort data collection.

Most patients carry a known *PTEN* P/LP variant, identified through Sanger sequencing or multigene next-generation sequencing (NGS) panels. A comprehensive chart review was performed extracting demographic, medical and genetic data, including age, biologic sex, follow-up duration, *PTEN* variant status and type, phenotypic features, family history of PHTS, occurrence of malignant and pre-malignant lesions and pharmacologic therapies received. Upper and lower gastrointestinal (GI) endoscopy reports were reviewed for inflammatory findings, polyp burden and histology. *PTEN* pathogenic variants were documented by the precise Human Genome Variation Society nomenclature (HGVS) (Transcript NM_314.8). Variants were classified according to the ClinGen *PTEN* Expert Panel Specifications to the ACMG/AMP Variant Interpretation Guidelines. The study was approved by the Institutional review board in each medical center. Informed consent was waived.

### Statistical methods and data analysis

Data were summarized using standard descriptive statistics. Categorical variables are presented as frequencies and percentages, and continuous variables are reported as medians with IQR.

## Results

### Clinical characteristics

Sixty-two patients were included in the study; 37.1% were female, the median age at diagnosis was 30 years (IQR 19–46), median follow-up time was 5.5 years (IQR 3–15), and 80% had at least one first-degree relative with PHTS diagnosis. The most common benign manifestations included colonic polyps (40 patients), macrocephaly (33.8%), benign thyroid disease and mucocutaneous lesions (29% and 26% respectively). Among the 12 patients who did not carry pathogenic/likely pathogenic variant in *PTEN* gene, the diagnosis was based on a combination of major and minor criteria according to NCCN guidelines[10]. Demographic characteristics and clinical findings are depicted in Table 1 and Supplementary Table 1.

**Table 1:** Demographic and clinical characteristics of patients with PHTS

Characteristics	Study Cohort (n=62)
Female sex, n (%)	23 (37.1)
Age at data collection years, median, (IQR)	39.5 (28–52)
Age at diagnosis, years median, (IQR)	30 (19–46)
Median follow-up time, years median, (IQR)	5.5 (3–15)
First degree family member with PHTS, n (%)	50 (80.6)
Ethnicity	
Jewish, n (%)	55 (88.7)
Arab, n (%)	7 (11.2)
PHTS phenotype	
Thyroid gland manifestations	
Benign, n (%)†	18 (29.0)
Thyroidectomy, n (%)	6 (9.6)
Mucocutaneous manifestations µ	
Oral mucosa lesions, n (%)	15 (24.1)
Pigmented macules on penis, n (%)	11 (17.7)
Other, n (%)	23 (37.1)
Macrocephaly, n (%)	21 (33.8)
Developmental delay, n (%)	8 (12.9)
Vascular abnormalities, n (%) ¥	5 (8.1)
Benign urologic manifestations, n (%) £	8 (12.9)
Benign gynecologic manifestations, n (%) €	5 (8.1)
mTOR Treatment, n (%) PTEN Genotype	5 (12.9)
ACMG based Classification	
Pathogenic/likely pathogenic variant, n (%)	50 (80.5)
Vus, n (%) *	2 (3.2)
Variant type:	
Nonsense, n (%)	10 (16.1)
Missense, n (%)	27 (43.5)
Frameshift, n (%)	11 (17.5)
Splicing, n (%)	2 (3.2)

^†^**Benign thyroid manifestations** included thyroid nodules, Hashimoto’s thyroiditis, goiter, adenoma, and hyperthyroidism. Thyroidectomy refers to non-malignant indications only

µ **Benign mucocutaneous manifestations** included palmar hemangiomas, facial trichilemmomas, hemangiomas, pigmented nevi, papillomas, osteomas, and hyperpigmentation. **Oral mucosal lesions** included hamartomas, fibromas, and fibrosquamous papillomas.

**Macrocephaly** was defined as head circumference >2 SD above the mean, >97th percentile, or documented macrocephaly in the medical record without specified measurements.

¥ **Vascular abnormalities** included hemangiomas and chronic thromboembolic pulmonary hypertension.

£ **Benign urologic manifestations** included hydrocele and microlithiasis.

€ Benign gynecologic manifestations included ovarian cysts.

IQR, interquartile range; ACMG, American College of Medical Genetics and Genomics; VUS, variant of uncertain significance, PHTS, *PTEN *Hamartoma Tumor Syndrome, mTOR mechanistic Target of Rapamycin. PTEN, Phosphatase and Tensin Homolog

*Patients fulfilled the clinical diagnostic criteria for PHTS.

### Endoscopic findings

Full endoscopic and pathologic reports were available for 48 subjects (77.4%).Overall, 40 patients (64.5% of total cohort) underwent colonoscopy, and 22 patients (35.5%) underwent esophagogastroduodenoscopy. Patients without available endoscopic data were either under the recommended age threshold for screening initiation (e.g., age 35 years according to NCCN guidelines) or had procedures performed at outside institutions prior to specialized center follow-up. Most of the endoscopic findings were benign. One case of early-stage colorectal cancer was identified in a 44-year-old patient (*PTEN*: c.53A>T). Polyps were observed throughout the digestive tract, most commonly in the colon (64.5%). In the colon, fourteen patients (22.6%) had less than 10 cumulative polyps, 14 patients had 11–100 polyps, and 8 patients had over 100 polyps. The most common histological type was hyperplastic polyps (n=20, 32.3%), followed by hamartomatous (n=13, 21%) and adenomatous polyps (n=5, 12.5%). Two patients demonstrated ulcerative colitis–like colonic inflammation, and one patient was diagnosed with celiac disease based on duodenal inflammation and supportive serologic findings. Sixteen patients had 11–100 gastric polyps, mostly hyperplastic. Sixteen patients had small bowel polyps, most commonly hyperplastic polyps located in the duodenum. Endoscopic findings are detailed in Table 2.

**Table 2:** Endoscopic findings

Gastrointestinal findings		N patients (%)
Colon		40 (83.3)
Number of polyps	<10	14 (35.0)
	11–100	14 (35.0)
	>100	8 (20.0)
Types of polyps	Hamartomatous polyp	13 (32.5)
	Hyperplastic polyp	20 (50.0)
	Adenomatous polyp	5 (12.5)
	Inflammatory	4 (10.0)
	Ganglion cell	1 (2.5)
	Colon Inflammation	4 (10.0)
Malignancy	Colorectal malignancy	1 (2.5)
Gastric		22 (45.8)
Number of polyps	<10	6 (27.2)
	11–100	16 (72.2)
	>100	0
Types of polyps	Hamartomatous polyp	6 (9.7)
	Hyperplastic polyp	14 (63.6)
	Inflammatory	4 (18.1)
Other	Gastritis	14 (63.6)
Small intestine		
polyps	Hyperplastic	8 (16.6)
	Inflammatory	8 (16.6)

^*^Percentages calculated from 48 patients with available endoscopic reports

### Malignancy rates

A total of 38 patients (61%) had at least one malignancy, diagnosed at a median age of 45 years. Among them, 5 patients (13.1%) had two distinct cancers, and 3 patients (7.8%) had three different cancers. The most frequent malignancy was breast cancer, occurring in 13 patients which represents 56.5% of female participants (13/23) at a median diagnosis age of 47 years (IQR 25–58); three of these patients had bilateral breast cancer. Thyroid cancer was the second most common malignancy, identified in 7 patients (11.3%) at a median age of 36.5 years (IQR 11–58). Central nervous system tumors were diagnosed in 4 patients and included soft-tissue skull-base tumors, dysplastic cerebellar gangliocytoma, and astrocytoma. Additional malignancies included renal cell carcinoma in 4 patients (6.4%), colon cancer in 1 patient (1.6%), and melanoma in 1 patient (1.6%) (Table 3).

**Table 3:** Neoplastic presentation

Malignancy	n (%)	Median age (y), IQR
Total	38 (61.3)	45 (30–50)
Thyroid	7 (11.3)	36 (19 – 46)
Breast: unilateral, bilateral	13 (50*), 3(13*)	47 (37 – 50)
Renal cell carcinoma	4 (6.4)	48 (47 – 50)
Endometrium	4 (17.3*)	44 (22–61)
Brain	4 (6.4)	19 (17–25)
Soft tissue	3 (4.8)	25 (18–38)
Gastrointestinal stromal tumor (GIST)	1 (1.6)	52
Colorectal	1 (1.6)	45
Melanoma	1 (1.6)	25

^*^Percentages were calculated out of the 23 women included in the study

### Atypical observations

Three subjects (4.8%) were diagnosed with soft tissue tumors:

A 58-year-old Ashkenazi Jewish woman with a history of breast cancer diagnosed at age 25, axillary osteosarcoma at age 38, and chest wall liposarcoma at age 47. PHTS was diagnosed at the age of 52 (pathogenic *PTEN* variant c.1034T>G; p.Leu345Arg). Surveillance endoscopy revealed multiple duodenal hypeplastic polyps (Patient #5). A 20-year-old Arab male with typical PHTS phenotype and a pathogenic *PTEN* variant (c.395G>T; p.Gly132Val) was diagnosed with abdominal desmoid tumor at the age of 18. (patient #12). Lastly, A 39-year-old Ashkenazi Jew female who presented in childhood with typical phenotype and a pathogenic *PTEN* variant. She was diagnosed with ovarian germ cell tumor at the age of 7 and soft tissue cancer at the base of the skull at the age of 30 (patient #17). All unique clinical findings are detailed in Table 4.

**Table 4:** Unique Observations and Genomic Profiles

Patient number	Genetic Variant	Typical PHTS manifestations	Co-occurring Observations (age of diagnosis)
*Unique PHTS clinical observations*			
#5	c.1034T>G p.(Leu345Arg)	Breast cancer; Duodenal hyperplastic polyps	osteosarcoma (38) Bilateral chest wall liposarcomas (47)
#12	c.395G>T p.(Gly132Val)	Macrocephaly; penile hyperpigmentation; GI polyps	Abdominal desmoid tumor (18)
#17	c.350dup p.(Asn117LysfsTer9)	Macrocephaly; thyroid goiter; colonic polyps; papillary thyroid cancer	Ovarian germ cell tumor (7) Skull base soft tissue malignancies (30)
*Unique PTEN genotype*			
#13	c.445C>T p.(Gln149Ter) PTEN germline mosaicism	Mucosal papillomatosis and papillary thyroid cancer premalignant uterine polyp	

### Mosaicism

One Ashkenazi Jewish female presented at the age of 47 with typical features of PHTS: mucosal papillomatosis and papillary thyroid cancer pre-malignant uterine polyp, despite the high probability of PHTS, genetic testing that included blood-based *PTEN* gene Sanger sequencing and subsequent *PTEN* promoter sequencing did not reveal any disease-causing variant. *PTEN* NGS of multiple tissue samples showed a variable expression of the *PTEN* pathogenic variant (c.445 C>T P.Q149X) as follows: 3% mosaicism in blood, 6% in saliva, 59% in endometrial polyp and 87% in thyroid lesion which suggest *PTEN* germline mosaicism. These findings along with the typical clinical manifestations established the diagnosis of PHTS. (Table 4).

### Unique PHTS treatment: mTOR inhibitors

Five patients (8%) were treated with the mTOR inhibitor sirolimus. Subject #2, who presented with a high burden of colonic polyps (> 100 polyps), was treated with sirolimus at a dose of 2 mg/day, and a reduction in polyp burden was noted on follow-up colonoscopies performed 1 and 2 years after treatment initiation. The remaining four patients were treated for large thyroid cysts, multinodular goiter, thyroid nodules, and astrocytoma. Disease burden remained stable throughout follow-up, and no adverse effects were reported.

## Discussion

In this multicenter cohort, we characterized the diverse and distinctive clinical manifestations, diagnostic approach, and treatment responses of individuals with PHTS. The malignancy spectrum observed in our cohort was largely consistent with that previously reported in PHTS [11, 12]. Overall, 61% of patients developed at least one malignancy, most commonly breast (uni or bilateral), and thyroid cancer. Notably, we also identified soft-tissue malignancies, osteosarcoma, and germ cell tumors in three patients. These uncommon cancers are not typically associated with PHTS, suggesting a potentially broader tumor spectrum in this condition. Notably, *PTEN* mosaicism was identified in an individual exhibiting classic PHTS symptoms despite negative blood-based *PTEN* sequencing.

Our cohort reinforces the clinical predictors established in the Tan et al. prospective study, although the study population was different. While the Tan study identified a 9.5% mutation yield among 3,042 probands satisfying relaxed PHTS clinical criteria, 80.6% of our cohort carried pathogenic variant. Compared with clinically based *PTEN* negative individuals, carriers of P/LP variant in *PTEN* gene had substantially higher frequencies of macrocephaly and gastrointestinal polyposis, similar to our cohort [13].In addition to the typical phenotype, our data highlights the wide and rare presentation spectrum of PHTS to include rare manifestations. While PHTS is characterized by well-established benign and malignant manifestations, individuals undergoing intensive surveillance or targeted cohort ascertainment frequently present with additional phenotypic findings. It remains crucial to differentiate true PHTS-driven spectrum features from coincidental events or ascertainment bias. Recent cohort data by Yehia et al. expanded the candidate tumor spectrum in PTEN carriers, suggesting an elevated risk for soft-tissue sarcomas [14]. Comprehensive molecular characterizations are essential to confirm whether these additional clinical observations are mechanistic features of PHTS or coincidental background events.

Notably, although soft tissue cancers are rare in the general population, with a lifetime risk of approximately 0.3% according to 2021–2023 SEER (Surveillance, Epidemiology, and End Results) data, they were identified in 4.8% of patients in our cohort [11, 15].

This observed occurrence of soft tissue cancer is particularly noteworthy. Previous case reports have described several cases of soft tissue sarcomas among individuals diagnosed with *PHTS* which include Ewing sarcoma [12], breast fibrosarcoma and femoral osteosarcoma [16]. Recent cohort data demonstrate that individuals with pathogenic *PTEN* variant have a higher risk of soft tissue sarcomas at younger ages compared to the general population, and these malignancies may occur alongside other PHTS component cancers such as breast, thyroid, and endometrial cancers [14]. As to patient #5 who developed axillary sarcoma following breast cancer, while radiation therapy is a well-established risk factor for secondary sarcomagenesisn [17], we hypothesize that the co-existence of germline *PTEN* dysfunction and localized radiation exposure creates a synergistic, 'two-hit' effect. In this scenario, the underlying loss of *PTEN* tumor suppressor function provides a baseline genetic susceptibility, while radiation-induced DNA damage acts as a severe environmental trigger that accelerates malignant transformation.

The pathogenesis of soft tissue malignancies in PHTS is linked to the loss of tumor suppressor function, which disrupts PI3K/AKT/mTOR signaling and impairs genomic stability, thereby facilitating malignant transformation in mesenchymal tissues and formation of soft tissue malignancies [18]. Our observations, together with those reported by others, support the concept that *PTEN* deficiency may broaden susceptibility to soft-tissue malignancies in PHTS and underscore the need for increased clinical awareness of atypical or mesenchymal tumors in this population.

Similarly, germ cell tumors are not traditionally considered part of the PHTS tumor spectrum, but growing evidence suggests a biologically plausible association. *PTEN* loss activates the PI3K/AKT pathway, promoting germ cell proliferation, and experimental models show that *PTEN* suppression contributes to germ cell tumor growth and progression [19, 20]. Several reports describe patients with P/LP variant *PTEN* s who developed germ cell tumors, including pediatric ovarian germ cell and cases in which germ cell tumors and acute megakaryoblastic leukemia shared *PTEN* and TP53 variants [21, 22].

Together, these findings support the possibility that, although rare, germ cell tumors may represent an underrecognized component of the PHTS-associated tumor spectrum.

The diagnosis of PHTS is based on clinical phenotype and identification of pathogenic change in *PTEN* gene. However, diagnosis is not always straightforward. We described a subject who had a typical clinical presentation for PHTS however, both Sanger sequencing of the *PTEN* coding region and NGS *PTEN* as well as promoter sequencing were negative. After examining affected tissues, *PTEN* mosaicism was noted and the diagnosis of PHTS was made. Several cases of mosaicism have been reported. One involved a 40-year-old woman with PHTS and Lhermitte-Duclos disease. Standard *PTEN* testing on blood DNA was negative, but a frameshift *PTEN* variant (c.767_768delAG) was later identified at full heterozygous levels in skin fibroblasts and cerebellar tumor tissue, and at ~25% in colonic and endocervical mucosa, confirming somatic mosaicism [23]. Similarly, two unrelated patients with juvenile polyposis, macrocephaly, intellectual disability, and hyperpigmented macules—consistent with BRRS—were reported. One carried a 10q23 deletion (*PTEN*, BMPR1A, KLLN) in buccal mucosa, while the other showed high-level mosaicism for a 10q23.2q23.3 deletion and a somatic *PTEN* mutation in thyroid tissue, with low-level mosaicism in blood and buccal samples [24]. These findings highlight the importance of the clinical pretest probability that relies on PHTS characteristic phenotype and that *PTEN* mosaic expression can be the cause for blood based germinal testing. While this case of constitutional mosaicism highlights a key diagnostic pitfall, its prevalence in PHTS remains unclear. Given that up to 35% of clinically diagnosed patients lack germline P/LP variant, the theoretical potential for tissue-based testing is high, though the actual yield remains undetermined [25].As advanced genetic testing becomes more accessible, tissue-based analysis is likely to identify further cases and gain clinical significance.

Vascular anomalies are increasingly recognized as an integral, though heterogeneous, component of the PHTS phenotypic spectrum, largely attributed to the up-regulation of the PI3K/Akt/mTOR pathway resulting from *PTEN* dysfunction, which alters normal angiogenesis. While literature commonly describes complex vascular malformations, including fast-flow arteriovenous malformations (AVMs) and deep intramuscular hemangiomas, our cohort primarily demonstrated localized hemangiomas. Notably, we also observed a rare manifestation of chronic thromboembolic pulmonary hypertension (CTEPH) [26].

We further describe our observations regarding the use of mTOR inhibitor (Sirolimus) in 5 patients.

While Sirolimus is not FDA- approved specifically forPHTS, its use is supported by mechanistic rationale and clinical data. As *PTEN* deficiency results in dysregulated activation of the PI3K–Akt–mTOR pathway, driving excessive cell growth and hamartoma formation characteristic of *PTEN-*related disorders, Sirolimus, through inhibition of mTOR complex 1, attenuates downstream signaling involved in protein synthesis and cell cycle regulation. By suppressing mTOR-mediated cellular proliferation, sirolimus provides a biologically rational therapeutic approach for mitigating disease manifestations in patients with *PTEN* loss [27]. mTOR inhibitor has shown clinical benefit in the management of vascular abnormalities and selected malignant manifestations, as well as regression of gastrointestinal lesions [5, 28].

In our cohort, sirolimus was not administered as a standard first-line therapy, but rather as an off-label, patient-tailored systemic intervention under the close monitoring of gastroenterology specialists working at a dedicated high-risk clinic. This targeted therapy was reserved for selected cases who had severe, progressive disease phenotypes and was administered in addition to standard endoscopic surveillance**.** This included cases with an exceptionally high burden of colonic polyps **(**Subject #2, with >100 polyps) and large, progressive thyroid nodules.

A reduction in polyp burden was observed in one patient, while the remaining treated patients demonstrated overall disease stabilization, with a favorable safety profile. Although the small sample size and retrospective design preclude definitive conclusions regarding efficacy, these findings strongly align with a recent clinical trial by Stanich et al., which also demonstrated a reduction in colon polyp burden with sirolimus in PHTS patients, thereby reinforcing the therapeutic potential and value of further prospective evaluation of sirolimus in this setting [29].

This study has several limitations that should be acknowledged. First, its retrospective design inherently limits data completeness and uniformity. Clinical, endoscopic, and pathologic evaluations were not performed according to a standardized protocol across the six participating centers, which may have introduced inter-institutional variability in phenotypic documentation, endoscopic surveillance intervals, and histological reporting.

Second, the cohort size was relatively small, reflecting the rarity of PHTS, and limits the statistical power to detect genotype–phenotype correlations or associations between specific *PTEN* variant classes and clinical outcomes, including malignancy risk and gastrointestinal burden. Consequently, analyses were primarily descriptive, and causal inferences cannot be drawn. In addition, the cohort is heterogeneous in both age and clinical presentation, which further complicates efforts to identify consistent patterns or establish meaningful genotype–phenotype correlations.

Despite these limitations, this multicenter cohort provides detailed phenotypic, endoscopic, and oncologic characterization of adult patients with PHTS, including rare manifestations, mosaicism, and real-world experience with mTOR inhibition. Notably, our study highlights significant clinical diversity across the adult lifespan. By capturing a broad age spectrum, we were able to observe distinct differences in disease expression between younger adults and older cohorts, particularly regarding the progressive severity and accumulation of symptoms over time. Importantly, patients were followed for a prolonged duration in specialized tertiary referral clinics with expertise in hereditary cancer syndromes, allowing for comprehensive longitudinal assessment.

In summary, our cohort underscores the marked clinical complexity and phenotypic variability of PHTS, as well as the risk of rare malignancies. Recognition of the potential unique phenotype and diagnostic challenges are important for PHTS surveillance. Future prospective studies with larger cohorts will be essential to refine surveillance guidelines, evaluate therapeutic options, and further clarify the natural history of this rare but clinically significant syndrome.

## Supplementary Information

Below is the link to the electronic supplementary material.

Supplementary Material 1

Supplementary Material 2

## Data Availability

No datasets were generated or analysed during the current study.
