# Supplementary material for: The clinical and phenotypic spectrum of PTEN hamartoma tumor syndrome: a retrospective cohort study
Source: Fam Cancer. 2026 Sep 17;25(4):88. doi: 10.1007/s10689-026-00604-w (PMC13585824; doi:10.1007/s10689-026-00604-w)
Supplement: Supplementary file 1 [file 10689_2026_604_MOESM1_ESM.docx]

| **Section** | **STROBE item** | **Reported on** |
| --- | --- | --- |
| Title/Abstract | Study design indicated in title/abstract | Title, Abstract |
| Introduction | Background/rationale | Introduction paragraphs 1–4 |
| Introduction | Objectives | Final paragraph of Introduction |
| Methods | Study design | Methods paragraph 1 |
| Methods | Setting | Methods paragraph 1 |
| Methods | Participants | Methods paragraph 1 |
| Methods | Variables | Methods paragraph 2 |
| Methods | Data sources/measurement | Methods paragraph 2 |
| Methods | Bias | Limitations section in Discussion |
| Methods | Study size | Entire cohort included; Methods |
| Methods | Quantitative variables | Statistical Methods section |
| Methods | Statistical methods | Statistical Methods and Data Analysis |
| Results | Participants | Results – Clinical characteristics |
| Results | Descriptive data | Table 1 |
| Results | Outcome data | Tables 2–4 |
| Results | Main results | Results sections |
| Discussion | Key results | Discussion paragraph 1 |
| Discussion | Limitations | Limitations section |
| Discussion | Interpretation | Discussion |
| Discussion | Generalizability | Discussion/Conclusion |
| Other information | Funding | Funding statement |
